# SFSSClass: an integrated approach for miRNA based tumor classification

**DOI:** 10.1186/1471-2105-11-S1-S22

**Published:** 2010-01-18

**Authors:** Ramkrishna Mitra, Sanghamitra Bandyopadhyay, Ujjwal Maulik, Michael Q Zhang

**Affiliations:** 1 Machine Intelligence Unit, Indian Statistical Institute, Kolkata, India; 2 Department of Computer Science and & Engineering, Jadavpur University, Kolkata, India; 3 Watson School of Biological Sciences, Cold Spring Harbor Laboratory, Cold Spring Harbor, NY 11724, USA; 4 MOE Key Laboratory of Bioinformatics and Bioinformatics Division, TNLIST, Tsinghua University, Beijing 100084, China

## Abstract

**Background:**

MicroRNA (miRNA) expression profiling data has recently been found to be particularly important in cancer research and can be used as a diagnostic and prognostic tool. Current approaches of tumor classification using miRNA expression data do not integrate the experimental knowledge available in the literature. A judicious integration of such knowledge with effective miRNA and sample selection through a biclustering approach could be an important step in improving the accuracy of tumor classification.

**Results:**

In this article, a novel classification technique called *SFSSClass *is developed that judiciously integrates a biclustering technique SAMBA for simultaneous feature (miRNA) and sample (tissue) selection (*SFSS*), a *cancer-miRNA *network that we have developed by mining the literature of experimentally verified cancer-miRNA relationships and a classifier uncorrelated shrunken centroid (USC). *SFSSClass *is used for classifying multiple classes of tumors and cancer cell lines. In a part of the investigation, poorly differentiated tumors (PDT) having non diagnostic histological appearance are classified while training on more differentiated tumor (MDT) samples. The proposed method is found to outperform the best known accuracy in the literature on the experimental data sets. For example, while the best accuracy reported in the literature for classifying PDT samples is ~76.5%, the accuracy of *SFSSClass *is found to be ~82.3%. The advantage of incorporating biclustering integrated with the *cancer-miRNA *network is evident from the consistently better performance of *SFSSClass *(integration of SAMBA, *cancer-miRNA *network and USC) over USC (eg., ~70.5% for *SFSSClass *versus ~58.8% in classifying a set of 17 MDT samples from 9 tumor types, ~91.7% for *SFSSClass *versus ~75% in classifying 12 cell lines from 6 tumor types and ~82.3% for *SFSSClass *versus ~41.2% in classifying 17 PDT samples from 11 tumor types).

**Conclusion:**

In this article, we develop the *SFSSClass *algorithm which judiciously integrates a biclustering technique for simultaneous feature (miRNA) and sample (tissue) selection, the *cancer-miRNA *network and a classifier. The novel integration of experimental knowledge with computational tools efficiently selects relevant features that have high intra-class and low inter-class similarity. The performance of the *SFSSClass *is found to be significantly improved with respect to the other existing approaches.

## Background

A family of ~22 nucleotide noncoding RNAs termed microRNA (miRNA) has been identified in eukaryotic organisms ranging from nematode to human [1-3]. MiRNAs regulate the expression of other genes by binding to complementary sites in the target messenger RNA (mRNA) through mRNA degradation or translational repression [4]. Increasing evidences indicate that miRNAs are key regulators of various fundamental biological processes such as cell cycle, cell growth and differentiation, apoptosis and embryo development, etc [5]. For example let-7 family of miRNAs identified in *C. elegans*, Drosophila, Zebrafish or Human [6-8] have important roles for terminal differentiation in normal embryonic development, temporal upregulation etc. In let-7 mutants, stem cells can fail to exit the cell cycle and terminally differentiate at the correct time [6], so that they continue to divide which is an indication of cancer.

Recent studies indicate that many miRNAs, referred to as onco/tumor suppressor miRNAs, are involved in the development of various human malignancies [9-11]. Differential expression of miRNAs contributes to carcinogenesis by promoting the expression of proto oncogenes or by inhibiting the expression of tumor suppressor genes [12,13]. Recently miRNA expression profiling data is being used for predicting the diagnostic categories of tissue samples including cancer versus non-cancer, multiclass tumor samples, etc. Based on a variation of the biological factors (such as tissue types, time points, etc.), a microarray expression data set can be made up of intra-class and inter-class samples [14]. The intra-class samples correspond to a common biological factor whereas inter-class samples possess different factors. To enhance the prediction accuracy it is important to identify the features (miRNAs) and samples (tissues), which are most informative with respect to the classification problem. The features and samples should be so selected that intra-class similarity increases and inter-class similarity decreases.

Motivated by this, here we develop *SFSSClass *algorithm which judiciously integrates a biclustering technique for simultaneous feature (miRNA) and sample (tissue) selection (*SFSS*), a newly constructed *cancer-miRNA *network and a classifier. The proposed method uses biclustering of miRNA expression profiling data to select features as well as samples/conditions relevant for classification. A bicluster provides a subset of the features that are co-expressed within a subset of the samples [15,16]. To increase the confidence that the selected features and samples are relevant, we integrate a *cancer-miRNA *network that we have constructed by mining the literature of experimentally verified cancer-miRNA relationships. This network lists all the miRNAs that have been found to be associated with different tumor types obtained from the literature. A lot of research has been devoted to the identification of specific miRNAs in specific cancers but such a comprehensive *cancer-miRNA *network based on differential expression patterns was still lacking in the literature. This network is not only useful in *SFSSClass*, it also throws up several new and interesting biological insights which are not evident in individual experiments, but become evident in the global graphical interface. For example, such a network can aid in the detection of cancer marker, identify hub miRNAs, reveal commonly altered regulatory pathways and also detect tissue specific (or cancer specific) miRNAs. These raise many unaddressed issues in miRNA research that have never been reported previously [17].

The novel integration of experimental knowledge and computational method efficiently selects relevant features that have high intra-class and low inter-class similarity. Thereafter, a supervised classifier USC is trained on the selected data in order to classify multiple classes of tumor tissues and cell lines. The experiments are conducted on the microarray data used in [9] and [18]. In a part of the investigation, poorly differentiated tumors (PDT) having non diagnostic histological appearance [9], but for which clinical diagnosis was established by anatomical context, are classified while training on more differentiated tumor (MDT) samples.

### Related work

In [9] a bead based miRNA expression profiling platform was used to measure the expression of 217 miRNAs in 334 tissue samples consisting of many different types of tumors some of which were poorly differentiated. The authors then used 68 samples having 11 tumor types to train a probabilistic neural network in order to classify the 17 PDT samples. They reported a classification accuracy of ~70.5%. This was much better when compared to the performance of the mRNA based classifier where they achieved ~5.9% classification accuracy. The work in [19] improved the accuracy to ~76.5% by proposing a classifier fusion approach using two bagged fuzzy k-NN classifiers with both mRNA and miRNA expression data (taking 40 genes from each). They also employed a feature selection technique called Relief-F [20]. When investigated on miRNA and mRNA data separately, the reported accuracies are ~70.5% and ~47.1%, respectively [19]. In [21] a comparative study is provided showing the classification accuracies of PDT samples obtained by executing different classifiers. The k-NN classifier (k = 1) obtained ~76.5% accuracy on discretized data but for continuous data a classification accuracy of ~58.8% is obtained by SVM and k-NN (k = 5). Here only four tumor classes are considered as training data (out of eleven available) since results with more number of classes was poorer.

## Methods

### Data

Three data sets (*Ds*_1_, *Ds*_2 _and *Ds*_3_) are considered for the experiments. For *Ds*_1_, training and test data are generated from miGCM_218.gct [9]. For *Ds*_2 _training and test data are generated from [18]. For *Ds*_3 _training data is generated from miGCM_218.gct and test data is generated from PDT_miRNA.gct [9]. Note that the test data set is totally independent in each experiment (i.e., it has not been used in anyway during training). For *Ds*_1_, 66 tumor samples are chosen from 9 MDT types among which 17 randomly chosen samples are considered for test data and the remaining are considered as training data. For *Ds*_2_, we have considered a total of 43 human cancer cell lines comprising central nervous system (CNS), colon, leukemia, melanoma, ovarian and renal tissue types. Another three tissue types such as breast, lung and prostate are excluded from the analysis as mentioned in [18], because breast and lung cancer cell lines have a lower intragroup correlations and for prostate, only two cell lines are available. Another cell line LOX IMVI of melanoma is excluded because it seems to be non melanotic and highly undifferentiated [22]. The full data set consisting of 627 probes, is first processed and filtered and select those probes which have expression values of ≥8, after log_2 _of raw expression value, in at least 10% of the cell lines. A total of 278 probes (miRNAs) have been selected. From 43 selected cell lines we have randomly chosen 12 cell lines as test set. For *Ds*_3_, 77 MDT samples from 11 distinct tumor types are chosen for training set and 17 PDT samples are chosen for the test set. The data is preprocessed, as suggested in [9], by filtering out those miRNAs whose expression values never exceed a minimal cutoff (≥7.25 on log2 scale) for all the samples. A detailed information regarding the data is given in Table 1 and in the Additional file 1.

**Table 1:** Selection of number of miRNAs, samples and classes from the training data in different stages of the experiment

Data Set	Original Data			After Pre-processing			After *SFSS*		
	
	miRNA	Sample	Class	miRNA	Sample	Class	miRNA	Sample	Class
*Ds*_1_	217	49	9	187	49	9	63	28	9
*Ds*_2_	627	31	6	278	31	6	77	22	6
*Ds*_3_	217	77	11	187	77	11	91	37	9

### Cancer-miRNA network

In order to globally observe and identify the miRNAs and associated cancer modules, generation of a *cancer-miRNA *network is crucial. As is evident, a particular type of cancer may be associated with the dysregulation of several distinct miRNAs and conversely dysregulation of one miRNA can be associated with several cancer types. In our previous work, generation of the *cancer-miRNA *network was based on the bipartite graph theoretic approach [17]. We formed a bipartite graph *G *= (*U*, *V*, *E*) where *U *is the set of cancer types, *V *is the set of miRNAs and (*u*, *v*) ∈ *E *iff *v *is differentially expressed or dysregulated in cancer type *u*. In other words, a bipartite graph based network model is constructed consisting of two disjoint sets of nodes where edges only exist between nodes from different sets. Here *U *is a set of 31 cancer types and *V *is a set of 192 cancer associated miRNAs. In order to develop the network, the differential expression patterns of experimentally verified human miRNAs in different cancer and normal tissue types obtained from extensive literature search are taken into account. Other relevant parameters that have been considered are location of the miRNAs at fragile sites and cancer associated genomic regions, epigenetic alteration of miRNA expression and abnormalities in miRNA processing target genes and proteins. The complete network is provided in a tabular form in Table S1 of Additional file 1.

### Classifier uncorrelated shrunken centroid

Uncorrelated shrunken centroid (USC) algorithm [23] is the robust version of the Shrunken Centroid (SC) algorithm [24], in which a sample is assigned to the class with the nearest average pattern. An instance is predictive of the class if at least one of its class centroids significantly differs from its overall centroid, termed as relative difference (*d*_*ik*_). The class centroid of an inatance *i *in class *k *is defined as the average expression level of that instance over all the samples in class *k*. Similarly, the overall centroid of an instance *i *is defined as the average expression level of that instance over all the experiments.

Let *x*_*ij *_= Expression level for instance *i *= 1, 2, ..., *p *and samples *j *= 1, 2, ..., *n*. Let number of classes = *K *and *C*_*k *_= Set of all *n*_*k *_samples in class *k*.

For *i*^*th *^instance overall centroid is,

and the class centroid of class *k *and instance *i *is,

*d*_*ik *_is standardized by the within class standard deviation of instance *i*(*s*_*i*_),

where *S*_0_= median value of *S*_*i*_*S *over all instance *i*.

The term 'significant' can be measured by shrinkage threshold Δ. If |*d*_*ik*_| > Δ then the instance with the corresponding class centroid is selected as relevant feature and used for classification. This can be stated as,

where  is referred to as shrunken relative difference. Instances with at least one positive shrunken relative difference  (over all classes *k*) are selected as relevant features. Based on the  the shrunken class centroid () can be defined as,

Now, the discriminant score for a new sample x* and class *k *can be defined as

where . The first term in the discriminant score represents the standardized square distance of x* to the shrunken class centroid and the second term represents a correction for the class prior probability.

Based on the minimum discriminant score sample x* is assigned to the class *k*.

In SC, a set of instances, *S*_Δ _is produced for a given shrinkage threshold Δ. As Δ increases, the number of relevant instances decrease since in this case the difference between the class centroid and the overall centroid of an instance needs to be larger for it to be considered as relevant. In USC, a set of redundant, correlated instances are further removed by computing the pairwise correlation for each pair of instances. If the pairwise correlation is greater than a correlation threshold *ρ *, the instance with the smaller relative difference is removed from the set of relevant instances. This way a set of relevant instances is generated for each shrinkage threshold Δ and correlation threshold *ρ*. This relevant instance set is then used for the classification. The USC algorithm is equivalent to the SC algorithm when *ρ *= 1 i.e. no correlated instances are removed from the list.

### SFSSClass: proposed classification method with simultaneous feature and sample selection

**Figure 1:**
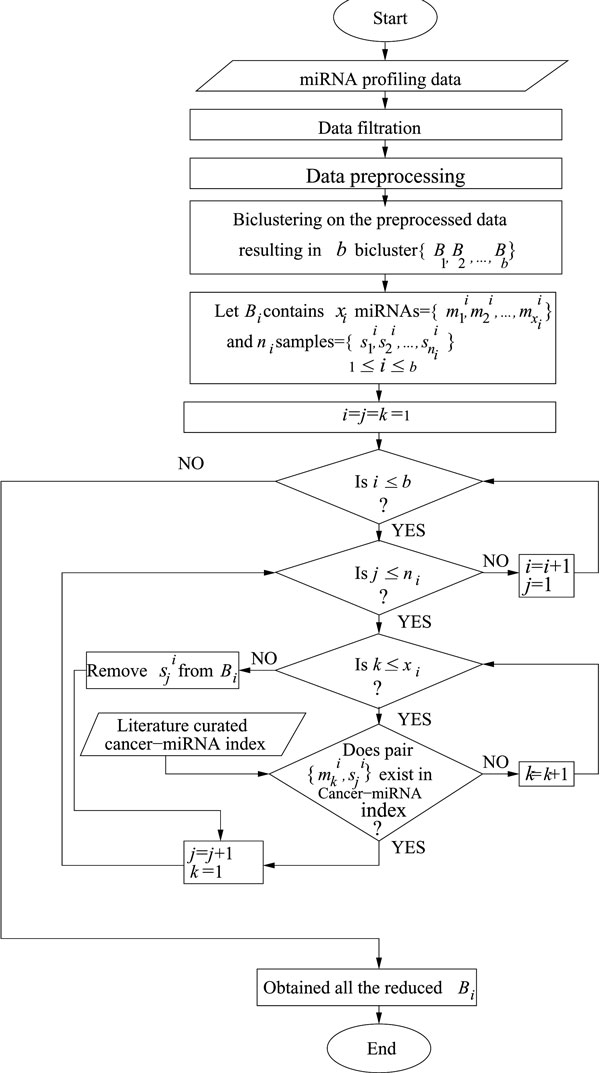
**A flow chart of SFSSClass, the proposed classification method with simultaneous feature and sample selection**.

Prediction accuracy of a classifier can be improved through the selection of relevant features and samples. The features are called relevant if these have high intra-class compactness and low inter-class similarity. In this regard we note that although expression data is available for a large number of miRNAs, only a small subset actually shows a similar expression pattern in a subset of tumor types due to their tissue specific regulatory nature. Thus, in this article we propose a technique called *SFSSClass *that uses biclustering for simultaneous feature and sample selection (*SFSS*). A flow chart of *SFSS *technique is provided in Figure 1.

The *cancer-miRNA *network is used in *SFSSClass *for selecting the relevant biclusters. We have used biclustering algorithm SAMBA [15] (a brief description of SAMBA is given in Additional file 1) on the preprocessed data set where the data is centered and normalized for each feature (miRNA), bringing the mean to 0 and standard deviation to 1. Among the obtained biclusters, we select those as potential ones which have atleast one miRNA that has existing biological evidence regarding it's correlation with at least one tumor sample. In other words, a bicluster is to be considered as potential if at least one cancer-miRNA association is present in the *cancer-miRNA *network. From a potential bicluster we choose only the relevant samples appearing in the *cancer-miRNA *network, but all the miRNAs are considered. The reason for considering all the miRNAs in a bicluster is that biological investigation has already revealed that genes belonging to the same cluster (or, bicluster) are likely to be co-regulated. Selected relevant miRNAs and samples are then used as the training set for the purpose of classification.

A set of relevant miRNAs (*S*_Δ_) is chosen based on shrinkage threshold Δ , where Δ and *S*_Δ _are inversely proportional. Again, a pairwise correlation for each pair of miRNAs (*g*_*i*_, *g*_*j*_) in *S*_Δ _is then computed for each Δ and it is determined whether this correlation is greater than a correlation threshold *ρ*. If so then the miRNA with smaller relative difference is removed from the set of relevant miRNAs. The optimal parameters (Δ and *ρ*) are determined from the results of the ten random fourfold cross validation. Based on the selected criteria the classification of the test set has been performed. We used publicly available tool EXPANDER version 3.2 for SAMBA http://acgt.cs.tau.ac.il/expander/expander.html and TIGR MeV version 3.1 [25] for executing the multiclass classifier USC. A detailed analysis of the results is described in the following section.

## Results and discussion

### Multi-class cancer classification using miRNA expression profiling data

#### Experiment 1(Exp_1_)

Here, a set of 17 MDT samples from 9 tumor types have been classified. In the proposed method, the classification is based on a training set of 63 miRNAs and 28 samples obtained by performing simultaneous feature and sample selection. We compared the performance of the proposed method with USC, k-NN^1 ^and k-NN^5^, and obtained a significantly better accuracy. Both USC and k-NN^1 ^obtained a prediction accuracy of ~58.8% and k-NN^5 ^obtained an accuracy of ~52.9% whereas *SFSSClass *obtained an accuracy of ~70.5% (see row *Exp*_1 _of Table 2). This underlines the importance of using the biclustering technique and *cancer-miRNA *network that is able to fetch the relevant miRNAs and samples prior to classification so that performance of the classifier is increased significantly. See Figure S1 and Figure S2 of Additional file 1 for the detailed analysis of the experiment.

**Table 2:** Number of selected features and samples, and comparison of classification accuracies obtained by different classifiers for Exp_1_: classification of multiclass MDT samples and Exp_2_: classification of multiclass cancer cell lines

Experiment	Classifier	After feature and sample selection			Classification accuracy (%)
			
		No. of miRNAs	No. of Samples	No. of Classes	
Exp_1_	SFSSClass	63	28	9	70.58
	USC	187	49	9	58.82
	kNN^1^	187	49	9	58.82
	kNN^5^	187	49	9	52.94

Exp_2_	SFSSClass	77	22	6	91.67
	USC	278	31	6	75
	kNN^1^	278	31	6	58.34
	kNN^5^	278	31	6	66.67

#### Experiment 2(Exp_2_)

Here, a set of 12 cell lines from 6 tumor types have been classified. The classification is based on a training set of 77 miRNAs and 22 samples obtained by performing simultaneous feature and sample selection. We compared the performance of the proposed method with USC, k-NN^1 ^and k-NN^5 ^and obtained a significantly better accuracy. In case of k-NN for k = 1 and k = 5 obtained prediction accuracies are of ~58.3% and ~66.7% respectively whereas USC obtained the prediction accuracy of 75%. Our method *SFSSClass *is found to outperform than the other methods and obtained a near optimal prediction accuracy of ~91.7% (see row *Exp*_2 _of Table 2). This again underlines the importance of selection of relevant features and samples using the biclustering technique in conjunction with the *cancer-miRNA *network prior to classification. See Figure S4 and Figure S5 of Additional file 1 for the detailed analysis of the experiment.

#### Classifying poorly differentiated tumors

**Figure 2:**
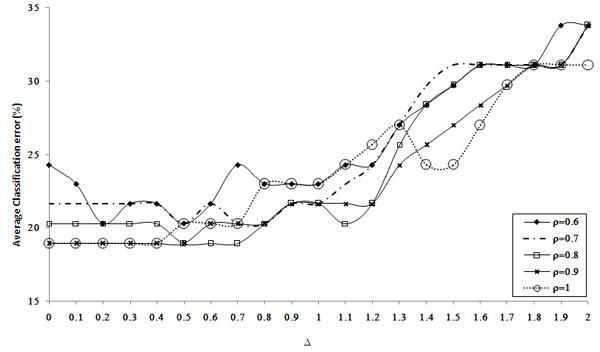
**Variation of the average classification error rate for different values of (Δ, ρ): selection of optimal parameter (Δ, ρ) based on ten random fourfold cross validation**.

In a part of the investigation we have classified the PDT samples based on a set of MDT training set. After performing simultaneous feature and sample selection from the training set, 91 miRNAs and 37 samples are selected from 9 tumor types, viz., colon, pancreas, kidney, bladder, prostate, ovary, uterus, lung and breast. In our biclustering experiment the miRNAs that are significantly dysregulated in mesothelioma or melanoma, did not appear in association with these two tissue types in any of the obtained biclusters. We have compared the prediction accuracies obtained by the proposed method with those reported previously in several literature including USC. The detailed results are shown in Table 3 and a brief description on various classifiers mentioned in the article is given in Table 1 of Additional File 2. The prediction accuracy is obtained based on the optimal parameters Δ = 0.3 and *ρ *= 0.9 for the USC and Δ = 0.1 and *ρ *= 0.9 for the proposed method as the minimum average classification error rate is obtained by the ten random fourfold cross validation using these parameters (for the detailed analysis of the experiment see Figure S3 of Additional file 1 and Figure 2 in the main text). From Table 3 it is observed that the proposed method provides much improved accuracy than any of the other approaches. Incorporation of the biclustering method and *cancer-miRNA *network improves the performance when USC algorithm is used (~82.3%) compared to the case without biclustering (~41.2%). This clearly shows the efficiency of the proposed method for extracting the relevant data through which more improved classification is possible.

**Table 3:** Number of selected features and samples, and comparison of classification accuracies obtained by different classifiers for the classification of multiclass PDT samples

Classifier	After feature and sample selection			Classification accuracy (%)
		
	No. of miRNAs	No. of Samples	No. of Classes	
SFSSClass	91	37	9	82.35
USC	187	77	11	41.17
DFL(Discretized data)	3	23	4	58.82
C4.5	3	23	4	52.94
RIP	3	23	4	35.29
NB	42	23	4	35.29
kNN^1^	42	23	4	47.05
kNN^5^	42	23	4	58.82
SVM	42	23	4	58.82
Classifier Fusion	40(miRNA)+40(mRNA)	68	11	76.47
Bagged Fuzzy kNN	40	68	11	70.58
PNN	173	68	11	70.58

## Conclusion

Recent evidences indicate that miRNAs have important roles in human malignancies and act as onco/tumor suppressor miRNAs. The cancer associated genomic regions, putative and experimentally verified target onco/tumor suppressor genes, significant over or under expression of the miRNAs in specific cancer cell lines are a few potential evidences of the involvement of miRNA in cancers. Limited work has been done towards revealing the fact that a number of miRNAs can control commonly altered regulatory pathways. However, this becomes immediately evident in the global graphical interface provided by the *cancer-miRNA *network proposed in our previous work [17]. In this article we develop the *SFSSClass *algorithm which judiciously integrates a biclustering technique for simultaneous feature (miRNA) and sample (tissue) selection, the *cancer-miRNA *network and a classifier. The performance of the *SFSSClass *is found to be significantly improved with respect to the other existing approaches. For example, while the best accuracy of classifying PDT samples obtained from [19] is ~76.5%, the accuracy of *SFSSClass *is found to be ~82.3%. The advantage of incorporating biclustering integrated with the *cancer-miRNA *network is evident from the consistently better performance of *SFSSClass *over USC (e.g., ~70.5% for *SFSSClass *versus ~58.8% in *Exp*_1_, ~91.7% for *SFSSClass *versus ~75% for USC in *Exp*_2 _and ~82.3% for *SFSSClass *versus ~41.2% for USC in classifying PDT samples).

Although the proposed approach is applicable to *cancer-miRNA *network, the concept of integrating domain knowledge (obtained through literature mining) based feature selection with classification may be useful in other Bioinformatics domains. For example, a very low prediction accuracy is obtained when classifying the PDT samples based on mRNA expression profiling data, ~ 5.9% in [9] and ~ 47.1% in [19]. In this context, judicious integration of *cancer-gene *network, biclustering and the classifier may improve the prediction accuracy. In future, specific information extracted from the *cancer-miRNA *network such as cancer specificity of miRNAs, hub miRNAs, over/under expressibility of miRNAs, etc., will be integrated with *SFSSClass *for more accurate prediction of tumor tissue origin.

## Competing interests

The authors declare that they have no competing interests.

## Authors' contributions

RM and SB performed all analysis and wrote the manuscript. UM and MQZ provided critical insights into the article. All authors read and approved the final manuscript.

## Supplementary Material

Additional file 1 **Appendix for "SFSSClass: An integrated approach for miRNA based tumor classification"**. The detailed information about cross validation result and chosen optimal parameters for both the USC and the proposed method are given in the figures s1 to S6. A complete list of all the miRNAs involved in different cancer types is provided in Table S1. The differential expression patterns of miRNAs in different tumor tissues along with a list of references (PubMed-indexed for MEDLINE or PMID) are also present in this table. The information is obtained by extensive literature search. Other relevant parameters that have been considered are location of the miRNAs at fragile sites and cancer associated genomic regions, epigenetic alteration of miRNA expression and abnormalities in miRNA processing target genes and proteins. Click here for file

Additional file 2 A brief description on various classifiers that have been used for classifying tumor samples. Click here for file
