# Supplementary material for: SFSSClass: an integrated approach for miRNA based tumor classification
Source: BMC Bioinformatics. 2010 Jan 18;11(Suppl 1):S22. doi: 10.1186/1471-2105-11-S1-S22 (PMC3009493; doi:10.1186/1471-2105-11-S1-S22)
Supplement: Additional file 1 — Appendix for "SFSSClass: An integrated approach for miRNA based tumor classification". The detailed information about cross validation result and chosen optimal parameters for both the USC and the proposed method are given in the figures s1 to S6. A complete list of all the miRNAs involved in different cancer types is provided in Table S1. The differential expression patterns of miRNAs in different tumor tissues along with a list of references (PubMed-indexed for MEDLINE or PMID) are also present in this table. The information is obtained by extensive literature search. Other relevant parameters that have been considered are location of the miRNAs at fragile sites and cancer associated genomic regions, epigenetic alteration of miRNA expression and abnormalities in miRNA processing target genes and proteins. [file 1471-2105-11-S1-S22-S1.pdf]

SUPPLEMENTARY *:*

SFSSClass: An integrated approach for miRNA based tumor classification

Ramkrishna Mitra [1], Sanghamitra Bandyopadhyay [1], Ujjwal Maulik [2] and Michael Q. Zhang [3, 4]

[1]Machine Intelligence Unit, Indian Statistical Institute, Kolkata, India
[2]Department of Computer Science & Engineering, Jadavpur University, Kolkata, India
[3]Cold Spring Harbor Laboratory, 1 Bungtown Road, Cold Spring Harbor, NY 11724, USA
[4]MOE Key Laboratory of Bioinformatics and Bioinformatics Division, TNLIST, Tsinghua University, Beijing 100084, China

---

We have considered microRNA exprepression profiling data from [1] and [2]. For the different experimental purposes mentioned in the original article the following files are used (All the data sets are available at the following address http://www.isical.ac.in/~rmitra_t/):

1 Data set extracted from Lu et al., 2005

1.1 Original Dataset

We have considered two data sets namely miGCM_218.gct and PDT_miRNA.gct from the original dataset of [1].

1.1.1 miGCM_218.txt

This dataset consists of 218 samples and 217 miRNAs. For Experiment 1 we have considered 66 Tumor samples from 9 tissue types viz. Colon, Pancreas, Kidney, Bladder, Prostate, Ovary, Uterus, Lung and Breast and for classifying poorly differentiated tumor (PDT) samples we have considered 77 Tumor samples from 11 tissue types viz. Colon, Pancreas, Kidney, Bladder, Prostate, Ovary, Uterus, Lung, Mesothelioma, Melanoma and Breast.

1.1.2 PDT_miRNA.txt

This dataset consists of 19 PDT samples and 217 miRNAs. For classifying PDT samples we have considered 17 samples as a test set from 4 tissue types viz. Colon, Ovary, Lung and Breast as mentioned in [1].

1.2 Preprocessed Dataset

The data is preprocessed as suggested in [1] by filtering out those miRNAs whose expression value never exceeds a minimal cut off (>7.25 on $\log_2$ scale) for all the samples.

1.2.1 Exp1: A set of More Differentiated Tumor (MDT) samples from 9 tissue types have been classified based on the following data sets and parameter.

(1) Without *SFSS* obtained dataset:
Without *SFSS* MDT train.txt
Without *SFSS* MDT test.txt
Without SFSS Sample label MDT train.txt
Without SFSS Sample label MDT test.txt

(2) After *SFSS* obtained dataset:
After *SFSS* MDT train.txt

After *SFSS* MDT test.txt
After SFSS Sample label MDT train.txt

(3) Optimal parameter (Δ, ρ) determination for the classifier USC is based on 10 random 4 fold cross validation:

(3a) Without *SFSS*:
Based on Figure S1 the chosen optimal parameter is $\Delta = 0.1$ and $\rho = 0.8$.

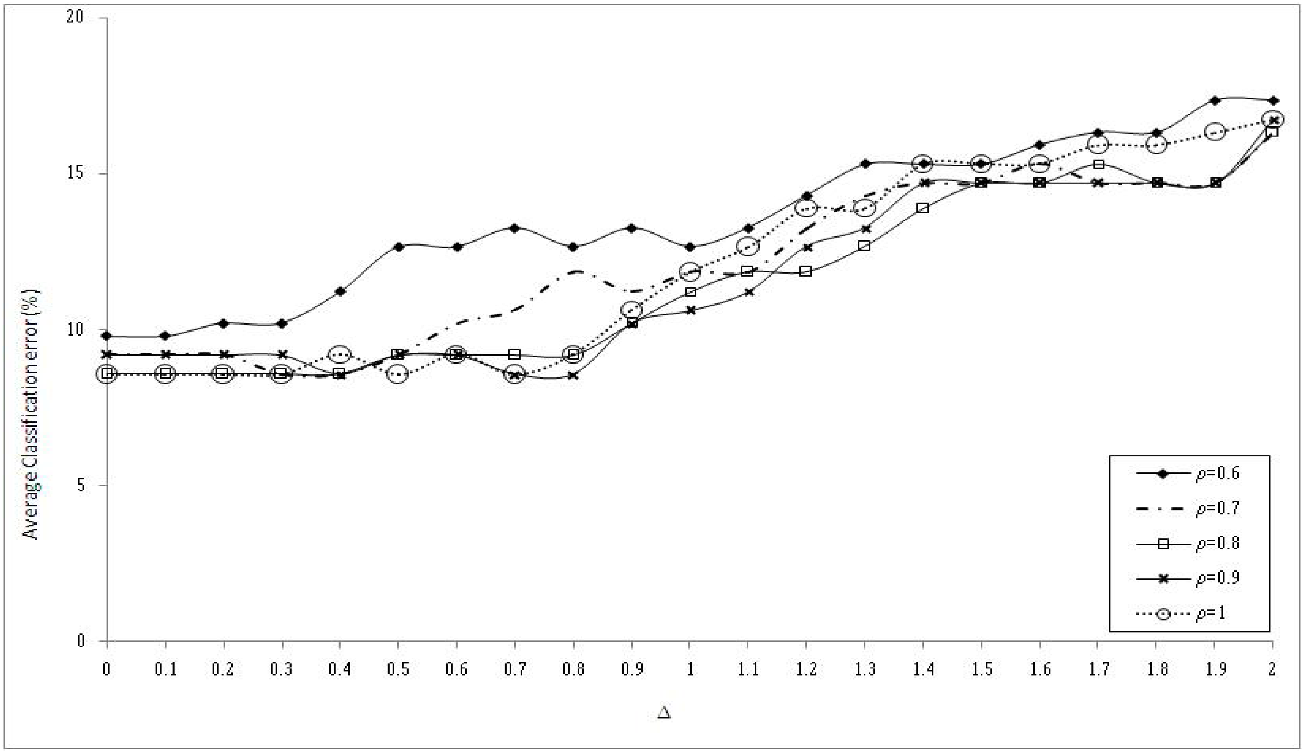

Fig S1. Selection of optimal parameter based on 10 random 4 fold cross validation.

(3b) After *SFSS*:
Based on Figure S2 the chosen optimal parameter is $\Delta = 0.4$ and $\rho = 0.9$

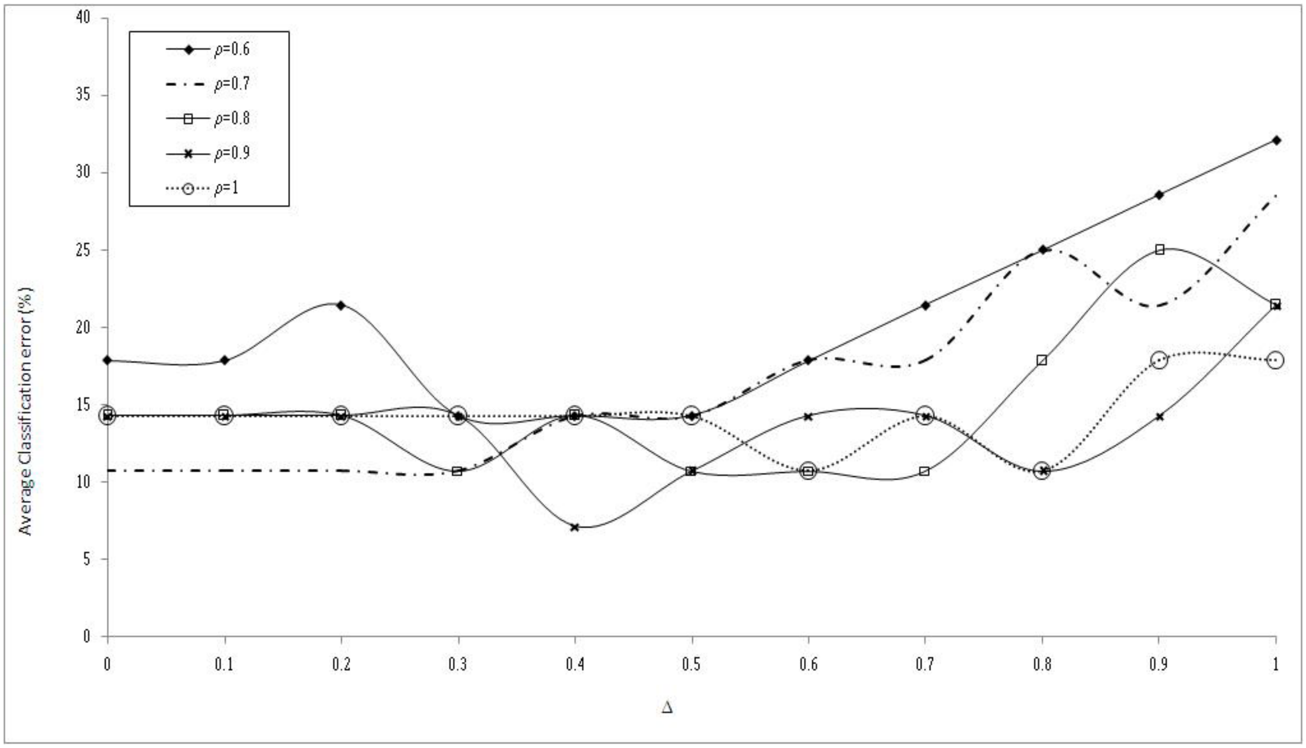

Fig S2. Selection of optimal parameter based on 10 random 4 fold cross validation.

1.2.3 Classifying PDT Samples: A set of Poorly Differentiated Tumor samples have been classified based on the following data sets and parameter.

(1) Without *SFSS* obtained dataset:
Without *SFSS* PDT train.txt
Without *SFSS* PDT test.txt
Without SFSS Sample label PDT train.txt
Without SFSS Sample label PDT test.txt

(2) After *SFSS* obtained dataset:
After *SFSS* PDT train.txt
After *SFSS* PDT test.txt
After SFSS Sample label PDT train.txt

(3) Optimal parameter (Δ, ρ) determination for the classifier USC is based on 10 random 4 fold cross validation:

(3a) Without *SFSS*:

Based on Figure S3 the chosen optimal parameter is $\Delta = 0.3$ and $\rho = 0.9$

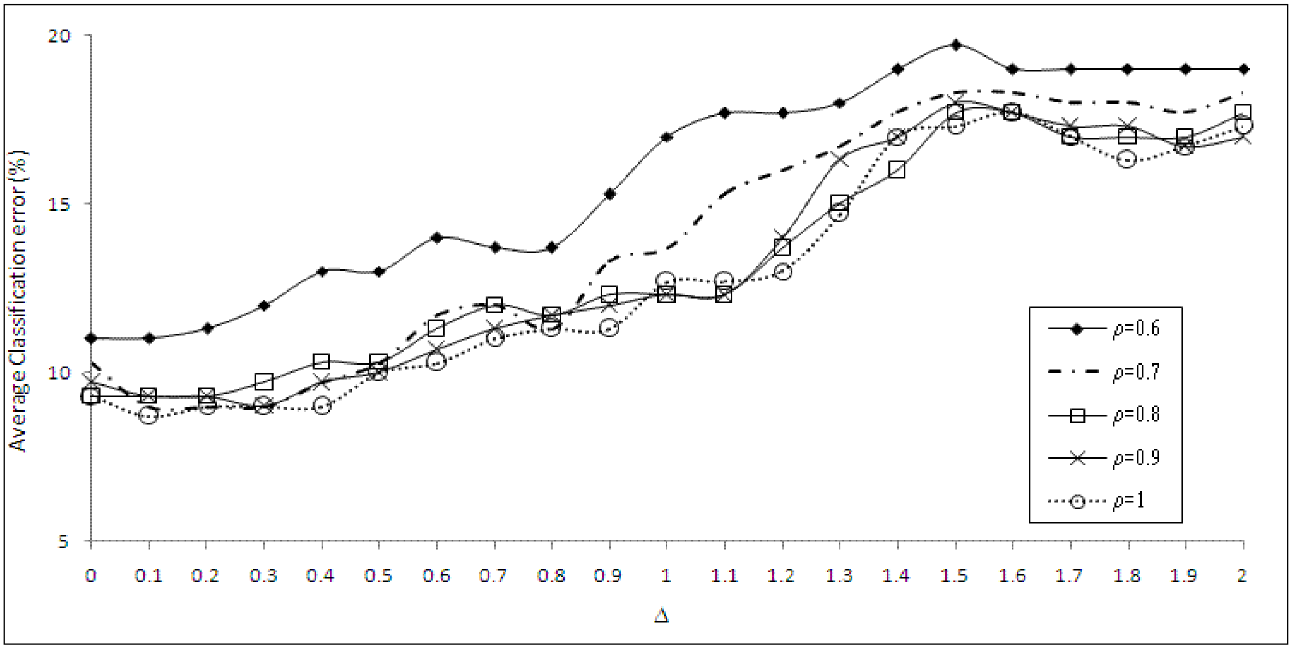

Fig S3. Selection of optimal parameter based on 10 random 4 fold cross validation.

2 Data set extracted from Blower et al., 2007

1.2.3 *Exp2*: A set of 12 cancer cell lines from 6 tumor types have been classified based on the following data sets and parameter.

(1) Preprocessed dataset (without *SFSS*):
Without *SFSS* NCI_60 train.txt
Without *SFSS* NCI_60 test.txt
Without *SFSS* Sample label NCI_60 train.txt
Without *SFSS* Sample label NCI_60 test.txt

(2) After *SFSS* obtained dataset:
After *SFSS* NCI_60 train.txt
After *SFSS* NCI_60 test.txt
After *SFSS* Sample label NCI_60 train.txt

(3) Optimal parameter (Δ, ρ) determination for the classifier USC is based on 10 random 4 fold cross validation:

(3a) Without *SFSS*:

Based on Figure S4 the chosen optimal parameter is $\Delta = 0.3$ and $\rho = 0.9$

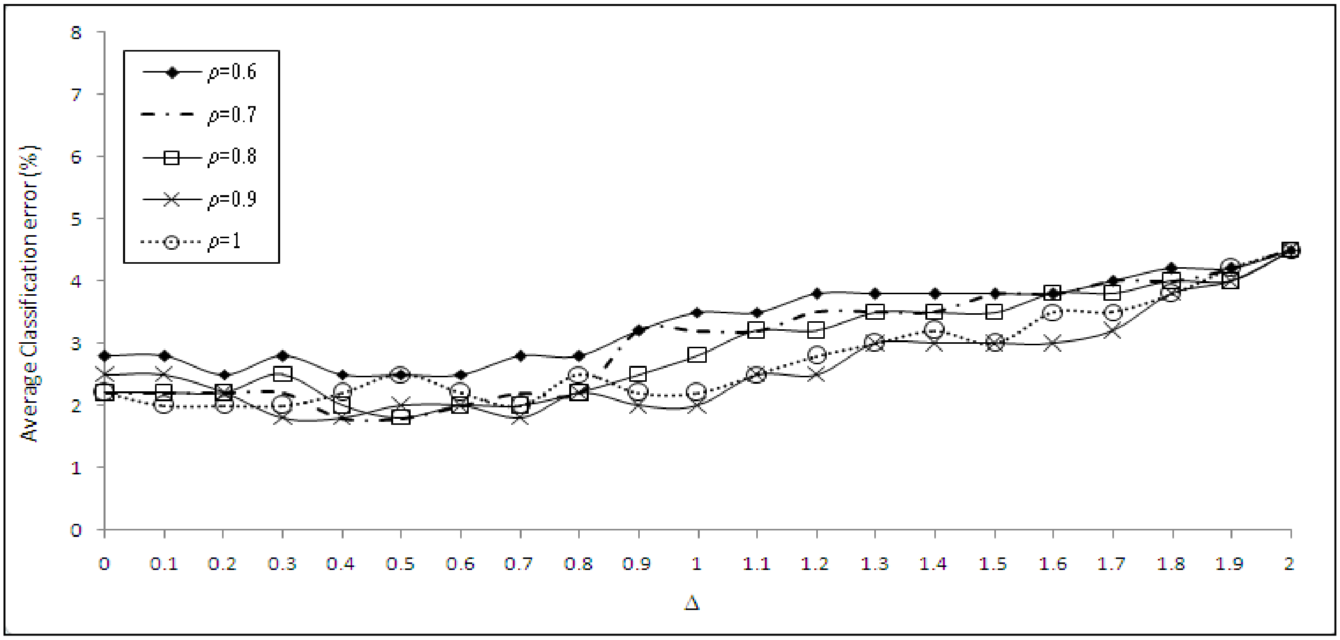

Fig S4. Selection of optimal parameter based on 10 random 4 fold cross validation.

(3b) After *SFSS*:

Based on Figure S5 the chosen optimal parameter is $\Delta = 0.1$ and $\rho = 0.9$

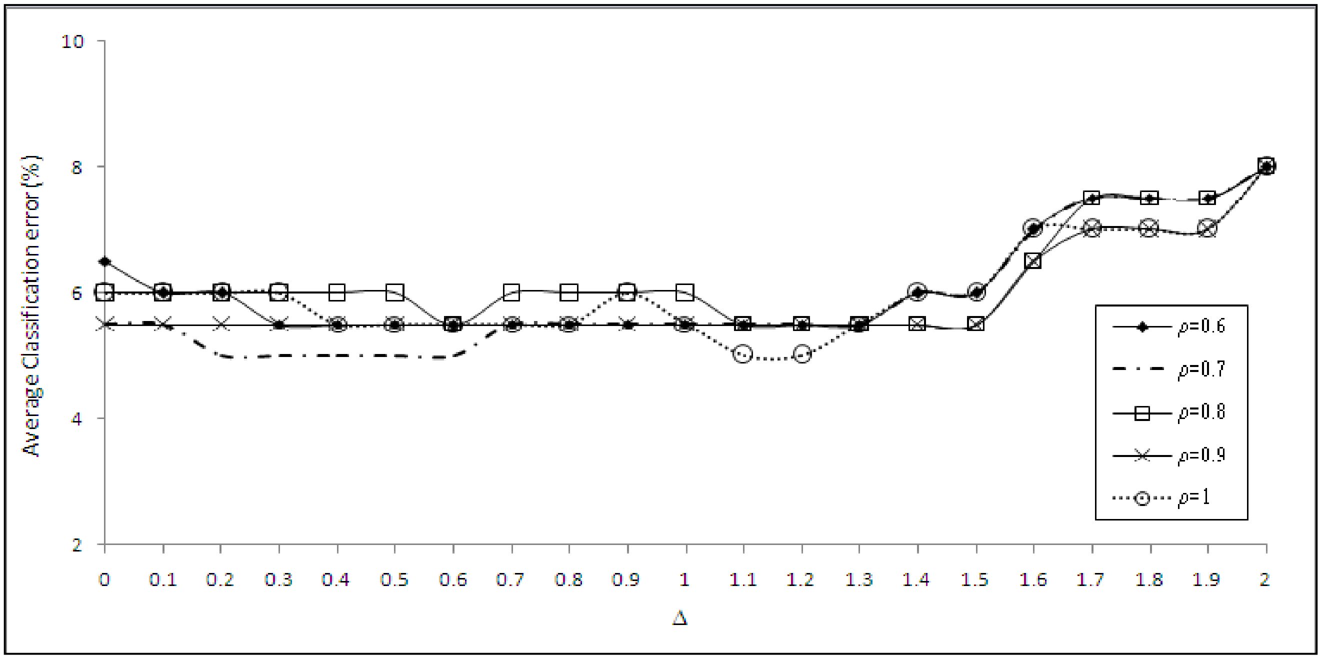

Fig S5. Selection of optimal parameter based on 10 random 4 fold cross validation.

3 Selection of optimal parameter

Performance of the classifier Uncorrelated Shrunken Centroid (USC) [3] is depend on the two threshold value – Shrinkage threshold (Δ) and Correlation threshold (ρ). It is a prerequisite for classification to select a smaller number of relevant features as the cost for classifying the patients sample is directly proportional to the number of features that should be tested to make the diagnosis. But only reduced number of features may not provide good prediction accuracy. Thus it is needed to determine the optimal parameter (Δ and ρ) in such a way that the classification accuracy is increased. To determine the optimal parameters, ten random fourfold cross validation has been performed on training set and observed for which parameter the average classification error rate is minimum. As can be seen from the fugures mentioned above the optimal parameter (Δ and ρ) is chosen based on the cross validation result. But only average classification error rate is not sufficient for *SFSSClass* algorithm to determine the optimal parameter. As in the proposed method a set of relevant features are already selected through Simultaneous Feature and Sample selection (*SFSS*) using Biclustering and *cancer-miRNA* network and it is assumed that these miRNAs have significant class information, thus further reduction of the number of the potential miRNAs is not desirable. As can be seen from Figure S5 that during cross validation minimum average classification error rate is obtained by a range of Δ values (0.2-0.6) and ρ = 0.7. But these values also reduced a significant number of miRNAs that are already selected by *SFSS* and assumed to have relevant class information. For classifying an independent test set based on the training samples it is not desirable to further reduce such relevant features. For example, though the above mentioned Δ and ρ values provide minimum average classification error rate during cross validation but provide a poor classification accuracy (75%) when classifying an independent test set compared to the other relevant parameters. Thus selection of the relevant number of features is also an important issue in *SFSSClass* algorithm. Selection of the number of miRNAs based on different values of (Δ and ρ) is shown in Figure S6(a) and S6(b).

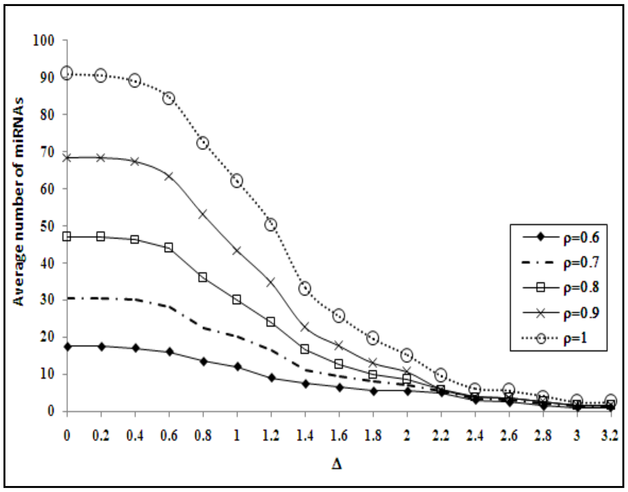

Fig. S6(a). For classifying PDT samples using MDT training set, during cross validation a number of miRNAs are selected based on different values of Δ and ρ.

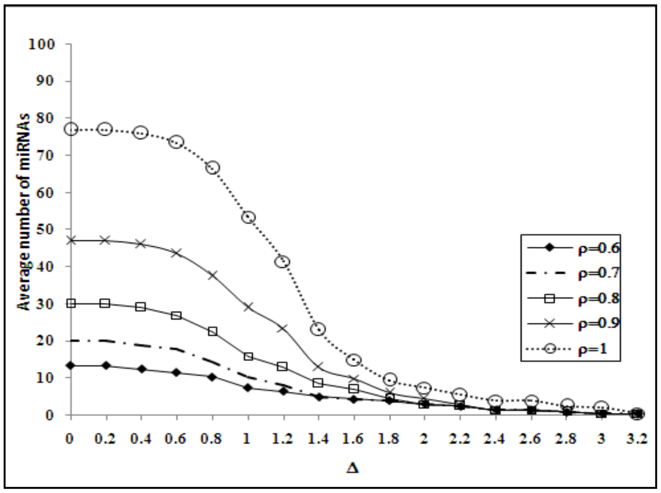

Fig. S6(b). In *Exp.*3, during cross validation a number of miRNAs are selected based on different values of Δ and ρ.

Figure S6(a) and S6(b) clearly shows for which parameter a significant number of miRNAs are reduced. For both the cases ρ= 0.6 and 0.7 and Δ > 0.4 always select a relatively reduced number of miRNAs. Beside this for all the experiments ρ= 1 is not considered as it provides a classification accuracy equivalent to Shrunken Centroid (SC) [4] algorithm.

4 Biclustering algorithm SAMBA: A short note

Groups of genes showing similar activity patterns under a specific subset of the experimental conditions, can be identified by a biclustering algorithm [5]. The concept of biclustering was first introduced by Hartigan in 1972 [6] and this technique was first implemented to gene expression data by Cheng and Church in the year 2000 [7]. Microarray data is stored in an n X p matrix M, which is defined by a set of rows (genes) $R = R_1, R_2, \ldots, R_n$ and a set of columns $C = C_1, C_2, \ldots, C_p$. An entry m_{gs} of M is a real value representing the expression level of gene g for sample s. A bicluster is a submatrix M_{GS} of M, where $G \subseteq R$ and $S \subseteq C$, having similar activity pattern. A bicluster consists of a subset of genes expected to be co-expressed within a subset of the conditions (belonging to that bicluster). In the proposed article we have considered a graph theoretic approach to biclustering combined with a statistical data model called SAMBA [8]. In this algorithm the gene expression matrix is considered as a bipartite graph $G = (U, V, E)$ where U is the set of conditions, V is the set of genes and $(u, v) \in E$ iff v responds in condition u. Biclusters produced by SAMBA is considered as a subgraph $H = (U', V', E')$ of G where V' is represented as sub-

set of genes that are coexpressed with a subset of conditions U'. A likelihood score assesses the significance of an observed subgraph . In SAMBA it has been shown how to assign weights to the vertex pairs of the bipartite graph. Quality of a computed bicluster in SAMBA is defined by the weight of the subgraph. The objective of SAMBA is to identify maximum weight subgraph assuming that the weight of the subgraph will correspond to its statistical significance. The most significant biclusters under the weighting scheme is equivalent to the selection of the heaviest subgraphs in the model bipartite graph. (In SAMBA, the genes with degree exceeding a threshold d are ignored.) This is biologically relevant since the genes that show high expression in many conditions contribute little to the bicluster. Moreover such genes are typically involved in several biological processes, and hence do not exhibit a specific effect as desirable in a bicluster.
In [8] two statistical models have been considered for the resulting bicluster. In the simpler model, it is assumed that all the values of all the genes in a given bicluster have changed relative to their normal level in the subset of the conditions that form the bicluster, without considering any kind of coherence of the values mg_s. Each value mg_s, can be represented either by the symbol A_1 (change) or A_0 (nochange) instead of its true values. An edge has been established between a gene and a condition if that gene has a changed expression level in that specific condition. No edge means no change. In the refined model, for each bicluster every two conditions must have the same or opposite effect for each of the genes. In this model the sign of the change is taken into account and is achieved by assigning a signal $C_{ij} \in \{-1,1\}$ to each edge of the graph and then looking for a bicluster (I, J) and an assignment $\tau : I \cup J \rightarrow \{-1,1\}$ such that $C_{ij} = \tau(i)\tau(j)$.
The algorithm finds K number of heaviest bicliques in the graph. In a post processing phase in order to perform a local improvement of the biclusters, SAMBA performs greedy addition or removal of vertices of the selected subgraph.

5 *CANCER-MIRNA* NETWORK

A complete list of all the miRNAs involved in different cancer types is provided in Table S1. The differential expression pattern of miRNAs in different tumor tissues along with a list of references (PubMed-indexed for MEDLINE or PMID) is also present in this table. The information is obtained from extensive literature search [9]. Other relevant parameters that have been considered are location of the miRNAs at fragile sites and cancer associated genomic regions, epigenetic alteration of miRNA expression and abnormalities in miRNA processing target genes and proteins.

Table S1: Tabular representation of Cancer-miRNA network (Last update - 13th August, 2009).

Cancer Type	miRNA	Expression	References (PMID)
Follicular_lymphoma	hsa-mir-19a	Up regulated/ Amplified	16885332, 14973191
Melanoma	hsa-let-7 family	Down regulated	18379589
CNS	hsa-let-7a	Down regulated	17363563
Hematologic	hsa-let-7a	Down regulated	17363563
Melanoma	hsa-let-7a	Deleted	18679415
Bladder (Urothelial)	hsa-let-7a-1	Deleted	14973191
Colon	hsa-let-7a-1	Down regulated	17965831
Lung	hsa-let-7a-1	Down regulated	17940623, 16712479
Uterus/Endometrial_cancer	hsa-let-7a-1	Up regulated	17243163
Breast	hsa-let-7a-2	Down regulated/ Deleted	17028596, 14973191
Cervical	hsa-let-7a-2	Deleted	14973191
Colon	hsa-let-7a-2	Down regulated	17965831
Lung	hsa-let-7a-2	Down regulated/ Deleted	17940623, 17096367, 14973191
Ovary	hsa-let-7a-2	Deleted	14973191
Uterus/Endometrial_cancer	hsa-let-7a-2	Up regulated	17243163
Colon	hsa-let-7a-3	Down regulated	17965831
Lung	hsa-let-7a-3	Down regulated	17028596
Uterus/Endometrial_cancer	hsa-let-7a-3	Up regulated	17243163
Colon	hsa-let-7b	Down regulated	17965831
Lung	hsa-let-7b	Down regulated	17940623, 17965831
Melanoma	hsa-let-7b	Up regulated	18379589
Uterus/Endometrial_cancer	hsa-let-7b	Up regulated	17243163

CNS	hsa-let-7c	Down regulated	17363563
Colon	hsa-let-7c	Down regulated	17965831, 17363563
Lung	hsa-let-7c	Down regulated/ Deleted	14973191
Uterus/Endometrial_cancer	hsa-let-7c	Up regulated	17243163
Bladder (Urothelial)	hsa-let-7d	Deleted	14973191
Colon	hsa-let-7d	Down regulated	17965831
Lung	hsa-let-7d	Down regulated	17965831
Uterus/Endometrial_cancer	hsa-let-7d	Up regulated	17243163
CNS	hsa-let-7e	Down regulated	17363563
Colon	hsa-let-7e	Down regulated	17965831
Hematologic	hsa-let-7e	Down regulated	17363563
Lung	hsa-let-7e	Down regulated	17965831
Uterus/Endometrial_cancer	hsa-let-7e	Up regulated	17243163
CNS	hsa-let-7f	Down regulated	17363563
Colon	hsa-let-7f	Down regulated	17363563
Bladder (Urothelial)	hsa-let-7f-1	Deleted	14973191
Lung	hsa-let-7f-1	Down regulated	15172979, 16712479
Uterus/Endometrial_cancer	hsa-let-7f-1	Up regulated	17243163
Colon	hsa-let-7f-2	Down regulated	17965831
Kidney	hsa-let-7f-2	Up regulated	17826655
Lung	hsa-let-7f-2	Down regulated	17940623, 17965831
Uterus/Endometrial_cancer	hsa-let-7f-2	Up regulated	17243163
Breast	hsa-let-7g	Deleted	14973191
CNS	hsa-let-7g	Down regulated	17363563
Colon	hsa-let-7g	Up regulated	16651716
Lung	hsa-let-7g	Down regulated	15172979
Uterus/Endometrial_cancer	hsa-let-7g	Up regulated	17243163
Colon	hsa-let-7i	Down regulated	17965831
Lung	hsa-let-7i	Down regulated	16712479
Uterus/Endometrial_cancer	hsa-let-7i	Up regulated	17243163
CNS	hsa-miR-1	Down regulated	17363563
Hematologic	hsa-miR-1	Down regulated	17363563
Breast	hsa-miR-100	Deleted	14973191
Cervical	hsa-miR-100	Deleted	14973191
HCC/Liver	hsa-miR-100	Up regulated	18307259
Lung	hsa-miR-100	Deleted	14973191
Ovary	hsa-mir-100	Deleted	14973191
Pancreas	hsa-miR-100	Up regulated	17473300
Pancreas	hsa-miR-100	Up regulated	17473300
Pancreas	hsa-mir-100-1/2	Up regulated	17473300
Breast	hsa-miR-101-1	Deleted	16530703
Lung	hsa-miR-101-1	Down regulated	16530703
Ovary	hsa-miR-101-1	Deleted	16530703
CNS	hsa-miR-103	Down regulated	17363563
Bladder (Urothelial)	hsa-miR-103-1	Up regulated	17826655
Colon	hsa-miR-106a	Up regulated	16461460
Lung	hsa-miR-106a	Up regulated	16530703
Ovary	hsa-mir-106a	Down regulated	18499237
Pancreas	hsa-mir-106a	Up regulated	16461460, 17442096
Prostate	hsa-miR-106a	Up regulated	16461460

CNS	hsa-miR-107	Down regulated	17363563
Colon	hsa-miR-107	Up regulated	16461460
Pancreas	hsa-miR-107	Up regulated	16461460
Stomach	hsa-mir-107	Up regulated	16461460
CNS	hsa-miR-10a	Up regulated	17363563
HCC/Liver	hsa-miR-10a	Up regulated	18307259
Hematologic	hsa-miR-10a	Down regulated	17363563
Breast	hsa-miR-10b	Down regulated	16466964, 17028596
HCC/Liver	hsa-miR-122	Down regulated	19296470
Lung	hsa-mir-123	Deleted	14973191
CNS	hsa-miR-124a	Down regulated	17363563
Lung	hsa-mir-124a-1	Down regulated	16530703
Lung	hsa-mir-124a-3	Down regulated	17028596
CNS	hsa-miR-125a	Down regulated	17363563
HCC/Liver	hsa-miR-125a	Down regulated	16331254, 17188425
Hematologic	hsa-miR-125a	Down regulated	17363563
Lung	hsa-miR-125a	Down regulated	16530703
Pancreas	hsa-miR-125a	Up regulated	17473300
Lung	hsa-miR-125a-prec	Down regulated	16530703
CNS	hsa-miR-125b	Down regulated	17363563
Colon	hsa-miR-125b	Down regulated	17363563
HCC/Liver	hsa-miR-125b	Up regulated	18307259
Hematologic	hsa-miR-125b	Down regulated	17363563
Lung	hsa-miR-125b	deletion	14973191
Breast	hsa-miR-125b-1	Down regulated	17028596, 16466964
Cervical	hsa-miR-125b-1	Deleted	14973191
Lung	hsa-miR-125b-1	deletion	14973191
Pancreas	hsa-mir-125b-1	Up regulated	17473300
Ovary	hsa-mir-125b-1	deletion/ Down regulated	14973191, 17875710
CNS	hsa-miR-126	Down regulated	17363563
Colon	hsa-miR-126	Down regulated	17363563
HCC/Liver	hsa-miR-126	Down regulated	16530703
Hematologic	hsa-miR-126	Down regulated	17363563
Lung	hsa-miR-126	Down regulated	16530703
CNS	hsa-miR-126*	Down regulated	17363563
Colon	hsa-miR-126*	Down regulated	17363563
HCC/Liver	hsa-miR-126*	Down regulated	16530703
Hematologic	hsa-miR-126*	Down regulated	17363563
Lung	hsa-miR-126*	Down regulated	16530703
Glioblastoma	hsa-miR-128	Down regulated	16039986
CNS	hsa-miR-128a	Down regulated	17363563
ALL	hsa-miR-128b	Up regulated	17891271
CNS	hsa-miR-128b	Down regulated	17363563
Colon	hsa-miR-128b	Up regulated	16461460
Lung	hsa-miR-128b	Up regulated	16461460
Pancreas	hsa-mir-128b	Up regulated	16461460
Colon	hsa-miR-130a	Down regulated	17363563
CNS	hsa-miR-132	Down regulated	17363563
Colon	hsa-miR-132	Down regulated	17363563
HCC/Liver	hsa-miR-132	Deleted	14973191

Hematologic	hsa-miR-132	Down regulated	17363563
CNS	hsa-miR-133b	Down regulated	17363563
Colon	hsa-miR-133b	Down regulated	17363563, 16854228
Hematologic	hsa-miR-133b	Down regulated	17363563
CNS	hsa-miR-134	Down regulated	17363563
Breast	hsa-miR-135-1	Deleted	14973191
Lung	hsa-mir-135-1	Deleted	14973191
CNS	hsa-miR-135a	Down regulated	17363563
Colon	hsa-miR-135b	Up regulated	16854228
CNS	hsa-miR-137	Down regulated	17363563
Melanoma	hsa-miR-137	Over expresed	18316599
CNS	hsa-miR-138	Down regulated	17363563
Nasopharyngeal/Nasal	hsa-miR-138-1	Deleted	14973191
CNS	hsa-miR-139	Down regulated	17363563
Colon	hsa-miR-139	Down regulated	17363563
CNS	hsa-miR-140	Down regulated	17363563
Colon	hsa-miR-140	Down regulated	17363563
Hematologic	hsa-miR-140	Down regulated	17363563
Lung	hsa-miR-140	Down regulated	16530703
Ovary	hsa-miR-140	Down regulated	17875710
Ovary	hsa-miR-141	Up regulated	17875710
Stomach	hsa-miR-141	Down regulated	19363643
CNS	hsa-miR-142-3p	Down regulated	17363563
CNS	hsa-miR-142-5p	Down regulated	17363563
Cervical	hsa-miR-143	Down regulated	17616659
CNS	hsa-miR-143	Down regulated	17363563
Colon	hsa-miR-143	Down regulated	16195701
Colon	hsa-miR-143	Down regulated	17363563
Hematologic	hsa-miR-143	Down regulated	17363563
Lung	hsa-mir-143	Down regulated	16530703
Prostate	hsa-miR-143	Down regulated	17616659
Breast	hsa-miR-145	Down regulated	16885332
Colon	hsa-miR-145	Down regulated	16195701, 16847880
Colon	hsa-miR-145	Down regulated	17363563, 16854228
HCC/Liver	hsa-miR-145	Down regulated	18307259
Hematologic	hsa-miR-145	Down regulated	17363563
Lung	hsa-mir-145	Down regulated	17028596
Ovary	hsa-miR-145	Downregulated	17875710
Prostate	hsa-miR-145	Down regulated	17028596
Breast	hsa-miR-146	Up regulated	16461460
Colon	hsa-miR-146	Down regulated	17363563
Hematologic	hsa-miR-146	Down regulated	17363563
Lung	hsa-mir-146	Up regulated	16530703
Pancreas	hsa-mir-146	Up regulated	16461460
Prostate	hsa-mir-146	Up regulated	16461460
Thyroid	hsa-miR-146b	Up regulated	17965831, 16885332
CNS	hsa-miR-150	Down regulated	17363563
Colon	hsa-miR-150	Down regulated	17363563
Hematologic	hsa-miR-150	Down regulated	17363563
Lung	hsa-miR-150	Up regulated	16530703

Colon	hsa-miR-152	Down regulated	17363563
Hematologic	hsa-miR-152	Down regulated	17363563
CNS	hsa-miR-154*	Down regulated	17363563
ALL	hsa-miR-155	Up regulated	16641092
Breast	hsa-miR-155	Up regulated	16461460, 16103050
Burkitts_lymphoma	hsa-mir-155	Up regulated	16466964, 18354490
Colon	hsa-miR-155	Up regulated	16461460, 16530703
Hematologic	hsa-miR-155	Down regulated	17363563
High-grade-lymphoma	hsa-mir-155	Up regulated	17487835
Lung	hsa-miR-155	Up regulated	17028596, 16530703
Pancreas	hsa-miR-155	Up regulated	17911294
Thyroid	hsa-miR-155	Up regulated	18270258
B-Cell-CLL	hsa-miR-15a	Down regulated	14973191
CLL	hsa-miR-15a	Deleted/Down regulated	14973191
Colon	hsa-miR-15a	Down regulated	17363563
Prostate	hsa-miR-15a	Down regulated	18931683
B-Cell-CLL	hsa-miR-15b	Down regulated	17234972
B-Cell-CLL	hsa-miR-16-1	Down regulated	17965831, 17531469
CLL	hsa-miR-16-1	Deleted/Down regulated	14973191
Prostate	hsa-miR-16-1	Down regulated	18931683
B-Cell-CLL	hsa-miR-16-2	Down regulated	12434020
Prostate	hsa-miR-16-2	Down regulated	18931683
B-Cell-CLL	hsa-miR-16a	Down regulated	14973191
Follicular_lymphoma	hsa-mir-17	Amplified	14973191
Lung	hsa-miR-17	Up regulated	16266980
Pancreas	hsa-mir-17*	Up regulated	16461460
CNS	hsa-miR-17-3p	Down regulated	17363563
Lung	hsa-mir-17-3p	Up regulated	16530703
Bladder (Urothelial)	hsa-miR-17-5p	Up regulated	17826655
Breast	hsa-miR-17-5p	Up regulated	16461460
Colon	hsa-miR-17-5p	Up regulated	16461460
Lung	hsa-miR-17-5p	Up regulated	16461460
Pancreas	hsa-miR-17-5p	Up regulated	16461460
Prostate	hsa-miR-17-5p	Up regulated	16461460
Follicular_lymphoma	hsa-mir-18	Amplified	14973191
HCC/Liver	hsa-miR-18	Up regulated	16331254
CNS	hsa-miR-181a	Down regulated	17363563
Glioblastoma	hsa-miR-181a	Down regulated	16039986, 18710654
AML	hsa-miR-181a-1	Deregulated	18450603
AML	hsa-miR-181a-2	Deregulated	18450603
CNS	hsa-miR-181b	Down regulated	17363563
Glioblastoma	hsa-miR-181b	Down regulated	16039986
ALL	hsa-miR-181b-1	Up regulated	17891271
Breast	hsa-miR-181b-1	Up regulated	16461460
Glioblastoma	hsa-miR-181b-1	Down regulated	18710654
Pancreas	hsa-mir-181b-1	Up regulated	16461460
Prostate	hsa-mir-181b-1	Up regulated	16461460
CNS	hsa-miR-181c	Down regulated	17363563
Glioblastoma	hsa-miR-181C	Down regulated	16039986
Lung	hsa-miR-181C-prec	Down regulated	17028596, 16530703

Colon	hsa-miR-183	Up regulated	16854228
Bladder (Urothelial)	hsa-miR-185	Up regulated	17826655
Kidney	hsa-miR-185	Up regulated	17826655
Thyroid	hsa-miR-187	Up regulated	18270258
HCC/Liver	hsa-miR-18-prec	Up regulated	16331254
CNS	hsa-miR-190	Down regulated	17363563
Colon	hsa-miR-190	Down regulated	17363563
CNS	hsa-miR-191	Down regulated	17363563
Colon	hsa-miR-191	Up regulated	16461460
Lung	hsa-miR-191	Up regulated	16461460
Pancreas	hsa-mir-191	Up regulated	16461460
Prostate	hsa-mir-191	Up regulated	16461460
Stomach	hsa-mir-191	Up regulated	16461460
Lung	hsa-mir-192	Down regulated	16530703
Thyroid	hsa-miR-192	Deleted	16530703
Lung	hsa-miR-192-prec	Down regulated	16530703
Thyroid	hsa-miR-192-prec	Deleted	16530703
CLL	hsa-miR-195	Up regulated	17891271
CNS	hsa-miR-195	Down regulated	17363563
HCC/Liver	hsa-miR-195	Down regulated	17188425, 16331254
Hematologic	hsa-miR-195	Down regulated	17363563
Lung	hsa-mir-195	Deleted	14973191
Pancreas	hsa-miR-196a-2	Up regulated	17473300
CNS	hsa-miR-196b	Up regulated	17363563
Hematologic	hsa-miR-196b	Down regulated	17363563
CNS	hsa-miR-197	Down regulated	17363563
Thyroid	hsa-miR-197	Up regulated	18270258
Uterus/Endometrial_cancer	hsa-miR-197	Down regulated	17243163
HCC/Liver	hsa-miR-198	Down regulated	18307259
Lung	hsa-miR-198	Down regulated	16530703
Colon	hsa-miR-199a	Down regulated	17363563
HCC/Liver	hsa-miR-199a	Down regulated	16331254
Hematologic	hsa-miR-199a	Down regulated	17363563
Ovary	hsa-miR-199a	Downregulated	17875710
Colon	hsa-miR-199a*	Down regulated	17363563
HCC/Liver	hsa-miR-199a*	Down regulated	16331254
Hematologic	hsa-miR-199a*	Down regulated	17363563
Pancreas	hsa-mir-199a-1	Up regulated	16461460
Prostate	hsa-mir-199a-1	Up regulated	16461460
Pancreas	hsa-miR-199a-2	Up regulated	17473300
Colon	hsa-miR-199b	Down regulated	17363563
Bladder (Urothelial)	hsa-miR-199b-prec	Down regulated	17470785
Lung	hsa-miR-199b-prec	Down regulated	16530703
Colon	hsa-miR-199-s	Down regulated	17363563
Hematologic	hsa-miR-199-s	Down regulated	17363563
Lung	hsa-mir-19a	Up regulated	16885332
Follicular_lymphoma	hsa-mir-19b-1	Amplified	14973191
HCC/Liver	hsa-miR-200a	Downregulated	16331254
Ovary	hsa-miR-200a	Up regulated	17875710
Ovary	hsa-miR-200b	Up regulated	17875710

Colon	hsa-miR-200c	Up regulated	18079988
Ovary	hsa-miR-200c	Up regulated	17875710
Esophageal	hsa-miR-203	Down regulated	18242245
Lung	hsa-miR-203	Up regulated	16530703
Ovary	hsa-miR-203	Up regulated	17875710
ALL	hsa-miR-204	Up regulated	17891271
CNS	hsa-miR-204	Down regulated	17363563
Colon	hsa-miR-204	Down regulated	17363563
Hematologic	hsa-miR-204	Down regulated	17363563
Esophageal	hsa-miR-205	Down regulated	18242245
Head & Neck	hsa-miR-205	Up regulated	17475218
Lung	hsa-miR-205	Up regulated	16530703
Ovary	hsa-miR-205	Up regulated	17875710
Breast	hsa-miR-205	Down regulated	19276373
Breast	hsa-miR-20a	Down regulated	16461460
Colon	hsa-miR-20a	Up regulated	16461460
Follicular_lymphoma	hsa-mir-20a	Amplified	14973191
Lung	hsa-mir-20a	Up regulated	16885332, 16266980
Pancreas	hsa-miR-20a	Up regulated	16461460
Prostate	hsa-miR-20a	Up regulated	16461460
Breast	hsa-miR-21	Up regulated	16103053, 16461460
Cervical	hsa-miR-21	Up regulated	17616659
Cholangiocarcinoma	hsa-miR-21	Up regulated	16762633, 19296468
CNS	hsa-miR-21	Up regulated	17363563
Colon	hsa-miR-21	Up regulated	16461460
Dif-fuse_large_B_cell_lymphoma	hsa-miR-21	Up regulated	17487835
Esophageal	hsa-miR-21	Up regulated	18242245
Glioblastoma	hsa-miR-21	Up regulated	16024602
HCC/Liver	hsa-miR-21	increased	18223217
Head & Neck	hsa-miR-21	Up regulated	17475218
Hematologic	hsa-miR-21	Down regulated	17363563
Lung	hsa-miR-21	Up regulated	16461460
Ovary	hsa-miR-21	Up regulated	17875710
Pancreas	hsa-mir-21	Up regulated	16461460
Prostate	hsa-mir-21	Up regulated	16461460, 19302977
Stomach	hsa-mir-21	Up regulated	16461460
Thyroid	hsa-miR-21	Up regulated	16365291
Uterus/Endometrial_cancer	hsa-miR-21	Up regulated	17243163
Breast	hsa-miR-210	induced	18316553
Lung	hsa-miR-210	Up regulated	16530703
Ovary	hsa-mir-210	Deleted	16530703
CNS	hsa-miR-212	Down regulated	17363563
HCC/Liver	hsa-miR-212	Deleted	14973191
Lung	hsa-miR-212	Up regulated	16530703
Uterus/Endometrial_cancer	hsa-miR-212	Down regulated	17243163
CNS	hsa-miR-213	Down regulated	17363563
Colon	hsa-miR-214	Down regulated	17363563
Lung	hsa-miR-214	Up regulated	16530703
Pancreas	hsa-miR-214	Up regulated	16461460

Prostate	hsa-miR-214	Up regulated	16461460
Stomach	hsa-mir-214	Up regulated	16461460
Lung	hsa-miR-216-prec	Down regulated	16530703
ALL	hsa-miR-218	Up regulated	17891271
Hematologic	hsa-miR-218	Down regulated	17363563
Breast	hsa-miR-218-2	Up regulated	16461460
Colon	hsa-miR-218-2	Down regulated	16461460
Lung	hsa-miR-218-2	Up regulated	16461460
Pancreas	hsa-mir-218-2	Down regulated	16461460
Prostate	hsa-miR-218-2	Down regulated	16461460
Stomach	hsa-mir-218-2	Down regulated	16461460
CNS	hsa-miR-219	Down regulated	17363563
Lung	hsa-miR-219-1	Down regulated	16530703
Colon	hsa-miR-22	Down regulated	17363563
HCC/Liver	hsa-miR-22	Deleted	14973191
Hematologic	hsa-miR-22	Down regulated	17363563
Lung	hsa-mir-220	Down regulated	16530703
Bladder (Urothelial)	hsa-miR-221	Up regulated	17826655
Colon	hsa-miR-221	Up regulated	16461460
Glioblastoma	hsa-miR-221	Up regulated	16039986
Pancreas	hsa-miR-221	Up regulated	16461460
Pancreas	hsa-miR-221	Up regulated	16461460
Stomach	hsa-mir-221	Up regulated	16461460
Thyroid	hsa-miR-221	Up regulated	17965831, 17468766
Thyroid	hsa-miR-222	Up regulated	16885332, 17468766
Bladder (Urothelial)	hsa-miR-223	Up regulated	17826655
CNS	hsa-miR-223	Down regulated	17363563
Colon	hsa-miR-223	Up regulated	16461460
Pancreas	hsa-miR-223	Up regulated	16461460
Prostate	hsa-miR-223	Up regulated	16461460
Stomach	hsa-mir-223	Up regulated	16461460
HCC/Liver	hsa-miR-224	Up regulated	16331254, 16331254
Lung	hsa-miR-224	Down regulated	16530703
Thyroid	hsa-miR-224	Up regulated	18270258
Bladder (Urothelial)	hsa-miR-23a	Up regulated	17826655
Colon	hsa-miR-23b	Down regulated	17363563
Hematologic	hsa-miR-23b	Down regulated	17363563
Uterus/Endometrial_cancer	hsa-miR-23b	Up regulated	17243163
Bladder (Urothelial)	hsa-miR-24-1	Deleted	14973191
Colon	hsa-miR-24-1	Up regulated	16461460
Pancreas	hsa-miR-24-1	Up regulated	16461460
Stomach	hsa-mir-24-1	Up regulated	16461460
Colon	hsa-miR-24-2	Up regulated	16461460
Lung	hsa-miR-24-2	Up regulated	16530703
Pancreas	hsa-miR-24-2	Up regulated	16461460
Stomach	hsa-mir-24-2	Up regulated	16461460
Pancreas	hsa-miR-25	Up regulated	16461460
Prostate	hsa-miR-25	Up regulated	16461460
Stomach	hsa-mir-25	Up regulated	16461460
CNS	hsa-miR-26a	Down regulated	17363563

Colon	hsa-miR-26a	Down regulated	17363563
Epithelial	hsa-miR-26a	Deleted	14973191
Hematologic	hsa-miR-26a	Down regulated	17363563
Nasopharyngeal/Nasal	hsa-miR-26a	Deleted	14973191
Epithelial	hsa-miR-26a1-prec	Deleted	14973191
Esophageal	hsa-miR-26a-1-prec	Deleted	16530703
Lung	hsa-miR-26a-1-prec	Down regulated	16530703
Bladder (Urothelial)	hsa-miR-26b	Up regulated	17826655
CNS	hsa-miR-26b	Down regulated	17363563
Kidney	hsa-miR-27	Up regulated	17826655
Uterus/Endometrial_cancer	hsa-miR-27a	Up regulated	17243163
Stomach	hsa-miR-27a	Up regulated	18789835
Bladder (Urothelial)	hsa-miR-27b	Deleted	14973191
Lung	hsa-miR-27b	Down regulated	16530703
Hematologic	hsa-miR-28	Down regulated	17363563
Kidney	hsa-miR-28	Up regulated	17826655
Colon	hsa-miR-297-3	Amplified	14973191
CNS	hsa-miR-299	Down regulated	17363563
Colon	hsa-miR-299	Down regulated	17363563
HCC/Liver	hsa-miR-299	Up regulated	18307259
CLL	hsa-miR-29a	Up regulated	17891271
Uterus/Endometrial_cancer	hsa-miR-29b	Down regulated	17243163
Breast	hsa-miR-29b-2	Up regulated	16461460
Colon	hsa-miR-29b-2	Up regulated	16461460
Lung	hsa-miR-29b-2	Down regulated	17028596
Pancreas	hsa-mir-29b-2	Up regulated	16461460
Prostate	hsa-mir-29b-2	Up regulated	16461460
CLL	hsa-miR-29c	Up regulated	17891271
CNS	hsa-miR-29c	Down regulated	17363563
Lung	hsa-mir-30a	Down regulated	17028596
Uterus/Endometrial_cancer	hsa-miR-30a	Up regulated	17243163
Lung	hsa-miR-30a-5p	Down regulated	17028596, 16530703
CNS	hsa-miR-30b	Down regulated	17363563
Ovary	hsa-miR-30b	Copy number gain	16754881
Colon	hsa-miR-30c	Up regulated	16461460
Pancreas	hsa-miR-30C	Up regulated	16461460
Prostate	hsa-miR-30C	Up regulated	16461460
CNS	hsa-miR-30d	Down regulated	17363563
Ovary	hsa-miR-30d	Copy number gain	16754881
CNS	hsa-miR-30e	Down regulated	17363563
Colon	hsa-miR-30e	Down regulated	17363563
CNS	hsa-miR-30e-3p	Down regulated	17363563
Colon	hsa-miR-30e-3p	Down regulated	17363563
Colon	hsa-miR-31	Up regulated	16854228
Colon	hsa-miR-32	Up regulated	16461460
Lung	hsa-miR-32	Down regulated	17028596
Pancreas	hsa-miR-32	Up regulated	16461460
Prostate	hsa-miR-32	Up regulated	16461460
Uterus/Endometrial_cancer	hsa-miR-32	Down regulated	17243163
Ovary	hsa-mir-320	Copy number loss	16754881

Breast	hsa-miR-320	Copy number loss	16754881
Melanoma	hsa-miR-320	Copy number loss	16754881
CNS	hsa-miR-321	Down regulated	17363563
CNS	hsa-miR-323	Down regulated	17363563
CNS	hsa-miR-324-3p	Down regulated	17363563
CNS	hsa-miR-324-5p	Down regulated	17363563
CNS	hsa-miR-328	Down regulated	17363563
Colon	hsa-miR-33	Deleted	16530703
Lung	hsa-miR-33	Down regulated	16530703
CNS	hsa-miR-330	Down regulated	17363563
ALL	hsa-miR-331	Up regulated	17891271
CLL	hsa-miR-331	Up regulated	17891271
CNS	hsa-miR-331	Down regulated	17363563
CNS	hsa-miR-338	Down regulated	17363563
CNS	hsa-miR-340	Down regulated	17363563
CNS	hsa-miR-342	Down regulated	17363563
Colon	hsa-miR-342	Down regulated	17363563
CNS	hsa-miR-345	Down regulated	17363563
CNS	hsa-miR-346	Down regulated	17363563
Breast	hsa-miR-34a	Down regulated	17028596
CLL	hsa-miR-34a	Up regulated	17891271
CNS	hsa-miR-34a	Down regulated	17363563
Lung	hsa-miR-34a	Down regulated	17028596
Breast	hsa-miR-34a-1	Deleted	14973191
Lung	hsa-miR-34a-1	Deleted	14973191
Breast	hsa-miR-34a-2	Deleted	14973191
Lung	hsa-miR-34a-2	Deleted	14973191
CNS	hsa-miR-361	Down regulated	17363563
CNS	hsa-miR-370	Down regulated	17363563
HCC/Liver	hsa-miR-370	Up regulated	18307259
Testicular_germ-cell_tumors	hsa-mir-372	hyper-expressed	17965831, 17189674
Testicular_germ-cell_tumors	hsa-mir-373	hyper-expressed	17965831, 17189674
Colon	hsa-miR-374	Down regulated	17363563
CNS	hsa-miR-375	Down regulated	17363563
Colon	hsa-miR-375	Down regulated	17363563
Pancreas	hsa-miR-375	Down regulated	17473300
CNS	hsa-miR-376a	Down regulated	17363563
Colon	hsa-miR-376a	Down regulated	17363563
Colon	hsa-miR-378	Down regulated	17363563
CNS	hsa-miR-379	Down regulated	17363563
Colon	hsa-miR-379	Down regulated	17363563
CNS	hsa-miR-382	Down regulated	17363563
CNS	hsa-miR-383	Down regulated	17363563
Colon	hsa-miR-422a	Down regulated	17363563
CNS	hsa-miR-422b	Down regulated	17363563
Colon	hsa-miR-422b	Down regulated	17363563
Colon	hsa-miR-424	Down regulated	17363563
CNS	hsa-miR-425	Down regulated	17363563
Lung	hsa-mir-9	Down regulated	16530703
Breast	hsa-miR-9-1	Increased, Copy number gain	17028596, 16754881

Pancreas	hsa-miR-92-2	Up regulated	16461460
Prostate	hsa-miR-92-2	Up regulated	16461460
Stomach	hsa-mir-92-2	Up regulated	16461460
Follicular_lymphoma	hsa-mir-92a-1	Up regulated/ Amplified	16885332, 17093929, 14973191
Lung	hsa-miR-95	Down regulated	16530703
Colon	hsa-miR-96	Up regulated	16854228
CNS	hsa-miR-98	Down regulated	17363563
Pancreas	hsa-miR-99	Up regulated	17473300
Colon	hsa-miR-99a	Down regulated	17363563
Hematologic	hsa-miR-99a	Down regulated	17363563
Lung	hsa-miR-99a	Deleted	14973191
CNS	hsa-miR-99b	Down regulated	17363563
Hematologic	hsa-miR-99b	Down regulated	17363563
Breast	Hsa-miR-328	Up regulated	19270061

REFERENCES

1. Lu J, Getz G, Miska E, Alvarez-Saavedra E, Lamb L, Peck D, Cordero AS, Ebert B, Mak R, Ferrando A, Downing J, Jacks T, Horvitz H, Golub T: Mi croRNA expression profiles classify human cancers. Nature 2005, 435:834–838.
2. Blower P, Verducci J, Lin S, Zhou J, Chung J, Dai Z, Liu C, Reinhold W, Lorenzi P, et al EK: MicroRNA expression profiles for the NCI-60 cancer cell panel. Mol Cancer Ther 2007, 6(5):1483–1491.
3. Yeung KY, Bumgarner RE: Multiclass classification of microarray data with repeated measurements: application to cancer. Genome Biol. 2003, 4:R83.
4. Tibshirani R, Hastie T, Narasimhan B, Chu G: Diagnosis of multiple cancer types by shrunken centroids of gene expression. Proc. Natl. Acad. Sci. USA 2002, 99:6567–6572.
5. Madiera SC, Oliviera AL: Biclustering algorithms for biologic-al data analysis: a survey. IEEE/ACM Trans.Comput.Biol.Bioinform 2004, 1:24–45.
6. Hartigan, JA: Direct clustering of a data matrix . J. Am Statistical Assoc. (JASA) 1972, 67: 123-129.
7. Cheng Y, Church GM: Biclustering of Expression Data. Proc. Eighth Intl Conf. Intelligent Systems for Molecular Biology 2000, 93-103.
8. Tanay A, Sharan R, Shamir R: Discovering Statistically Significant Biclusters in Gene Expression Data. Bioinformatics 2002, 18:S136–S144.
9. Bandyopadhyay S, Mitra R, Maulik U, Zhang MQ: Development of the Human Cancer MicroRNA Network. *BMC Silence* (*Accepted*).
