# Supplementary material for: SFSSClass: an integrated approach for miRNA based tumor classification
Source: BMC Bioinformatics. 2010 Jan 18;11(Suppl 1):S22. doi: 10.1186/1471-2105-11-S1-S22 (PMC3009493; doi:10.1186/1471-2105-11-S1-S22)
Supplement: Additional file 2 — A brief description on various classifiers that have been used for classifying tumor samples. [file 1471-2105-11-S1-S22-S2.pdf]

Table 1: A brief description on various classifiers that have been used for classifying tumor samples

Classifier	Bief Description	References
DFL	According to Discrete Function Learning (DFL) algorithm if the mutual information between a vector and the class attribute $I(X;Y)$ equls to the entropy of the class attribute $H(Y)$, then the class attribute is the function of the vector. Based on the mutual information and the class attribute, DFL algorithm is able to determine the most discriminatory feature vectors. The determined subset of features are supposed to contain all or most information of the class attribute. After the learning process, the DFL algorithm provides the classifiers as function tables which contain the most discriminatory feature vectors and the class attributes. The prediction is performed with the 1-Nearest Neighbor algorithm. The classifier selects the class value of the rule which has the minimum Hamming distance to the new sample as the predicted class value.	[1, 2]
C4.5	C4.5 is a decision tree learning algorithm. To classify a test instance, this algorithm generates a decision tree based on a set of attributes. At every step, the algorithm considers the attribute with the highest information gain and creates a decision based on that attribute to split the training set into one subset per discrete value of the feature, or two subsets based on a threshold comparison for continuous features. The process will continue recursively until all nodes are final. After generating the decision tree C4.5 prunes the tree in order to avoid over fitting based on some user specified setting.	[3]
RIPPER	Repeated Incremental Pruning to Produce Error Reduction (RIPPER) is the modified version of the rule learning algorithm IREP. It performs efficiently on large noisy data sets. From a set of pre-labeled instances it finds a set of rules that predict the class of earlier instances. In this algorithm the users are allowed to specify constraints on the learned if-then rules to add prior knowledge about the concepts in order to obtain more accurate hypothesis.	[4]
NB	The classifier Naive Bayes (NB) is based on the Bayes' theorem. Based on the assumption that the attributes are conditionally independent, given the class label y, the classifier estimates the class-conditional probability. The conditional independence assumption can be stated as follows: $P(X\|Y=y)=\prod_{i=1}^{d} P(X_i\|Y=y)$ where each attribute set $X = X_1, X_2, ..., X_d$ consists of d attributes. With the conditional independence assumption there is needed to estimate the conditional probability of each X_i, given Y. To classify a test data, the classifier computes the posterior probability for each class Y, stated as: $P(Y\|X)=\frac{P(Y)\prod_{i=1}^{d} P(X_i\|Y)}{P(X)}$ Posterior probabilities can be estimated by computing the product between the prior probability and the class-conditional probabilities. The prior probabilities of each class can be estimated by calculating fraction of training records that belong to each class.	[5]
kNN	k-Nearest Neighbor algorithm uses specific training instances to make predictions without considering an abstraction or model derived from data. This is an instance based learning algorithm that require a proximity measure in order to determine the similarity or distance between instances and a classification function. The classification function returns the predicted class of a test instance based on its proximity to other instances. The classifier makes their predictions based on local information. The classifier can produce arbitrarily shaped decision boundaries. The decision boundaries of the classifier have high variability because they depend on the composition of training examples. Increasing the number of nearest neighbors may reduce such variability. A test instance is classified by a majority vote of its k neighbors. Any ties can be broken at random. In binary classification problems, tied votes can be avoided by choosing k to be an odd number.	[6]
Bagged Fuzzy kNN	Fuzzy kNN is an extension of kNN where the crisp class labels have been replaced by soft labels, $l(v_i) \in [0,1]^c$. The method proposed by [8] has been used in the referred article that combining the soft labels of the kNN to compute the soft output label $l(x)$ for $x \in X$. Bagging is a classifier that generates multiple versions of the fuzzy kNN classifier. The soft class labels from these classifiers are then averaged to obtain the final output.	[7]
Classifier Fusion	In the referred article top ranked informative mRNAs and miRNAs are first selected using the feature selection algorithm proposed in [9]. Two Bagged Fuzzy kNN classifiers are then trained with mRNA and miRNA expression profiling data separately. For each test tissue sample, the decision of the two classifiers are aggregated by considering the decision of the classifier with higher confidence.	[7]
PNN	The probabilistic Neural network (PNN) algorithm can be categorized into the broad class of "nearest neighbor-like algorithms". The likelihood function of a given class is represented by PNN as the sum of identical, isotropic Gaussians. The Variance (σ) of the Gaussians is the only free parameter of the likelihood function. Rest of the terms in this function are determined directly from the training data. The likelihood function for class i can be stated as, $L_i(\vec{x})=\frac{1}{N_i(2\Pi\sigma)^{k/2}}\sum_{j=1}^{N_i} e^{-(\vec{x}-\vec{x}_j^i)^2/\sigma}$ and the conditional probability for class i is $P_i(\vec{x})=L_i(\vec{x})/\sum_{j=1}^{M} L_j(\vec{x})$ where the exemplars from class i be the k vectors $\vec{x}_j^i$ for $j=1,...,N_i$.	[10]
SVM	Support Vector Machine (SVM) is a supervised learning algorithm that achieves a high classification accuracy in many applications. For the data set that cannot be linearly separated in the input space, SVM can learn the classifier by transforming the input data into another higher dimensional feature space where it is easy to compute an accurate classification. The transformation of the feature space is achieved by the choice of a suitable kernel function. Given m training vectors $x_k \in R^n$, $k=1,...,m$ and a vector of labels $y \in R^m$ such that $y_k \in$ {1,-1}, the SVM in training learns a hyperplane (ω, b), optimally separating the items of the two classes, defined as: $\min_{\omega,b,\xi} \quad \frac{1}{2}\omega^T\omega + C\sum_{k=1}^{m}\xi_k$, subject to $yk(\omega^T\phi(x_k)+b) \geq 1-\xi_k$, $\xi_k \geq 0, k=1,...,m.$ The function ϕ maps the training data to a higher dimensional space and C is a penalty parameter on the training error. With a learned hyperplane (ω, b), a query vector x can be classified based on the decision value $f(x)$ or the SVM score: $f(x)=sign(\omega^T\phi(x)+b)$. We need a kernel function $k(x,x')=\phi(x)^T\phi(x')$ for mapping the data to a higher dimensional space. The query vector x is compared with the feature vector of each of the training instance using the kernel function in order to calculate the decision value $f(x)$. Given the expression of $f(x)$, the decision in which class x belongs to is taken into account.	[11, 12]

References

1. Zheng Y, Kwoh CK: **Cancer classification with microRNA expression patterns found by an information theory approach**. *Journal of computers* 2006, **1**:30–39.
2. Zheng Y, Kwoh CK: **Identifying simple discriminatory gene vectors with an information theory approach,**. *In Proceedings of the 4th Computational Systems Bioinformatics Conference, CSB 2005. IEEE Computer Society Press, 2005*:12–23.
3. Quinlan J: **C4.5: Programs for machine learning**. *Morgan Kaufmann* 1993.
4. Cohen WW: **Fast Effective Rule Induction**. *Machine Learning: Proceedings of the 12th International Conference (ML95), Morgan Kaufmann* 1995, :115–123.
5. Langley P, Iba W, Thompson K: **An analysis of bayesian classifiers**. *In National Conference on Artificial Intelligence* 1992, :223–228.
6. Aha DW, kibler D, Albert M: **Instance-based learning algorithms**. *Machine Learning* 1991, **6**:37–66.
7. Wang Y, Dunham MH, Waddle JA, McGee M: **Classifier Fusion for Poorly-Differentiated Tumor Classification using Both Messenger RNA and MicroRNA Expression Profiles**. *Proceedings of the 2006 Computational Systems Bioinformatics Conference (CSB 2006), Stanford, California* 2006.
8. Keller JM, Gray MR, Givens JA: **fuzzy k-nearest neighbor algorithm**. *IEEE Transactions on Systems, Man and Cybernetics* 1985, **15**:580–585.
9. Kononenko I: **Estimating attributes: analysis and extensions of relief**. *In Proceedings of the European conference on machine learning* 1994, :171–182.
10. Specht DF: **Probabilistic neural networks**. *Neural Networks* 1990, **3**:109–118.
11. Vapnik VN: **The Nature of Statistical Learning Theory**. *Berlin: Springer-Verlag* 1995.
12. Vapnik VN: **Statistical Learning Theory**. *Wiley-Interscience* 1998.
